# 

**Published:** 1867-03

**Authors:** 


### Dental Register.

EDITED BY

J. TAFT & G. WATT.

**MARCH 1867**

MONTHLY:

**THREE DOLLARS PER ANNUM, IN ADVANCE.**

CINCINNATI:
WRIGHTSON & CO., Printers, 167 WALNUT STREET.

*J. & C. R. TAFT, Dentists, 238 West Fourth St.*

